# Systematic Co-design of Tech Innovations for Aging in Place in a Smart World

**DOI:** 10.1093/geroni/igaf122.958

**Published:** 2025-12-31

**Authors:** Sajay Arthanat

**Affiliations:** University of New Hampshire, Durham, New Hampshire, United States

## Abstract

Rapid innovations in information communication technologies, home automation, robotics, wearables and digital health platforms offer tremendous promise in helping sustain the independence of older adults in the community. However, the design and intended application of these technologies are often not in sync with proven foundations of aging, consumer dispositions and skillset of older consumers. Dr. Arthanat will emphasize the significance of co-designing technological innovations with older adults based on a series of exploratory, multifactorial, and empirical research studies he has conducted over a span of a decade. The presentation will feature an ongoing project to co-design an AI-framework for socially assistive robots to provide personalized care for individuals with Alzheimer’s disease and related dementia at home

